# Supplementary material for: Prolonged Differentiation of Neuron-Astrocyte Co-Cultures Results in Emergence of Dopaminergic Neurons
Source: Int J Mol Sci. 2023 Feb 10;24(4):3608. doi: 10.3390/ijms24043608 (PMC9959280; doi:10.3390/ijms24043608)
Supplement: Supplementary file 1 [file ijms-24-03608-s001.zip › Supplementary Figure S1.pdf]

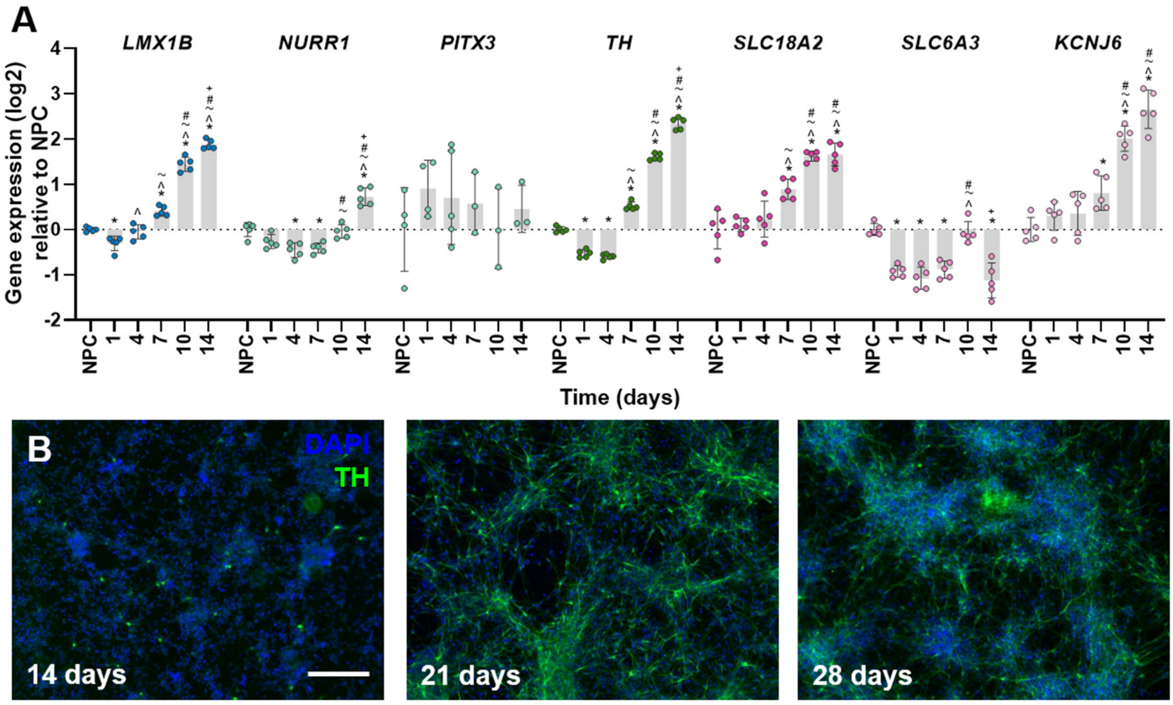

Supplementary Figure S1. Differentiation of dopaminergic neurons up to 14 days in culture. (**A**) Gene expression over time of a selection of dopaminergic markers for cell types relative to the neural progenitor cell (NPC) culture, ordered by expected timing of expression. For function of the markers, see description of Figure 1B. For gene expression of general neuronal markers the reader is referred to de Leeuw et al. (2022). (**B**) TH expression over time at 14, 21 and 28 days. Significance (adjusted *p*-value ≤ 0.05) is indicated relative to *: NPC, ^: 1 day, ~: 4 days, #: 7 days, +: 10 days. Full statistics can be found in Supplementary Table S2. Scale bar: 200 μm.
